# Advanced Backcross QTL Analysis of Fiber Strength and Fineness in a Cross between *Gossypium hirsutum* and *G. mustelinum*

**DOI:** 10.3389/fpls.2017.01848

**Published:** 2017-10-25

**Authors:** Baohua Wang, Zhimin Zhuang, Zhengsheng Zhang, Xavier Draye, Lan-Shuan Shuang, Tariq Shehzad, Edward L. Lubbers, Don Jones, O. Lloyd May, Andrew H. Paterson, Peng W. Chee

**Affiliations:** ^1^School of Life Sciences, Nantong University, Nantong, China; ^2^Plant Genome Mapping Laboratory, University of Georgia, Athens, GA, United States; ^3^Department of Crop and Soil Sciences, University of Georgia, Tifton, GA, United States; ^4^Engineering Research Center of South Upland Agriculture, Ministry of Education, Southwest University, Chongqing, China; ^5^Earth and Life Institute, Université catholique de Louvain, Louvain-la-Neuve, Belgium; ^6^Agricultural Research Division, Cotton Incorporated, Cary, NC, United States

**Keywords:** genetic diversity, introgression, marker-assisted breeding, fiber strength, fiber fineness

## Abstract

The molecular genetic basis of cotton fiber strength and fineness in crosses between *Gossypium mustelinum* and *Gossypium hirsutum* (Upland cotton) was dissected using 21 BC_3_F_2_ and 12 corresponding BC_3_F_2:3_ and BC_3_F_2:4_ families. The BC_3_F_2_ families were genotyped with simple sequence repeat markers from a *G. hirsutum* by *G. mustelinum* linkage map, and the three generations of BC_3_-derived families were phenotyped for fiber strength (STR) and fineness (Micronaire, MIC). A total of 42 quantitative trait loci (QTLs) were identified through one-way analysis of variance, including 15 QTLs for STR and 27 for MIC, with the percentage of variance explained by individual loci averaging 13.86 and 14.06%, respectively. Eighteen of the 42 QTLs were detected at least twice near the same markers in different generations/families or near linked markers in the same family, and 28 of the 42 QTLs were identified in both mixed model-based composite interval mapping and one-way variance analyses. Alleles from *G. mustelinum* increased STR for eight of 15 and reduced MIC for 15 of 27 QTLs. Significant among-family genotypic effects (*P* < 0.001) were detected in 13 and 10 loci for STR and MIC respectively, and five loci showed significant (*P* < 0.001) genotype × family interaction for MIC. These results support the hypothesis that fiber quality improvement for Upland cotton could be realized by introgressing *G. mustelinum* alleles although complexities due to the different effects of genetic background on introgressed chromatin might be faced. Building on prior work with *G. barbadense, G. tomentosum*, and *G. darwinii*, QTL mapping involving introgression of *G. mustelinum* alleles offers new allelic variation to Upland cotton germplasm.

## Introduction

This is the fourth report describing the interspecific *G. mustelinum* by *G. hirsutum* (Upland cotton) genetic map and mapping and introgressing quantitative trait loci (QTLs) of fiber quality traits from *G. mustelinum* into Upland cotton. In three previous papers, we reported the interspecific *G. hirsutum* by *G. mustelinum* genetic linkage map (Wang et al., 2016b), and described 24 QTLs for fiber elongation (Wang et al., 2016a,b) and 65 QTLs for fiber length traits (Wang et al., 2017). Some alleles from *G. mustelinum* improved fiber properties, demonstrating their potential value for improving fiber quality in Upland cotton breeding.

Here interspecific QTL mapping and introgression were reported for two important fiber quality traits, fiber strength (STR) and fineness measured in Micronaire (MIC). STR is determined as the necessary force breaking a fiber “beard.” Following the measurement of length by the Uster High Volume Instrument (HVI), the measurement of strength is performed on the same fiber beard with two sets of jaws clamping the beard by using a gauge length of 3.175 mm. The breaking force is measured directly and normalized with an assessment of the fiber mass from the optical sensor (in combination with the MIC value) to give the strength in cN/tex (Naylor et al., 2014). Fiber tenacity usually affects yarn tenacity more than any other properties of fiber at optimum yarn twist, and when fiber strength increases one cN/tex, yarn strength will increase about 0.5 cN/tex or even more. Generally bundle tenacity higher than 30 cN/tex is considered desirable (Estur and Knappe, 2007). Usually STR should be increased in cotton breeding programs.

MIC is a measure of the air permeability of compressed cotton fibers, which reflects both maturity in the development degree of cell walls and fiber fineness measured in linear density. A constant cotton fiber mass is compressed into a space with known volume. This compressed sample is used to measure air permeability, which is converted to appropriate numbers denoting MIC values. Generally MIC readings of 3.7–4.2 are premium, 3.5–3.6 or 4.3–4.9 are base, and 3.4-and-under or 5.0-and-higher are substandard and may result in a discounted price to the producer (http://www.cottoninc.com/fiber/quality/Classification-Of-Cotton/Classing-booklet.pdf). When the measurement comes in too low, the cotton is more susceptible to entangling around debris, which means too much of the good fiber will also be lost. When it is too high, it also causes problems since a coarser fiber negatively affects the spinning process, as well as overall quality is undesirable from the aspect of yarn evenness and spinning (Montalvo, 2005). The fineness determines how many fibers are present in the cross-section of a yarn of given thickness. More fibers available in the cross section of yarn generally results in stronger yarn, usually produced with finer fibers (Estur and Knappe, 2007). Additional fibers in the cross-section provide not only additional strength but also better evenness in the yarn. Many Upland cotton varieties have the problem of high MIC values currently, so it is necessary to breed varieties with comparatively low MIC or finer fiber.

This research aims to map QTLs for STR and MIC in a set of advanced-backcross *G. hirsutum* × *G. mustelinum* population. The QTLs mapped in this research will enhance our understanding of the molecular genetic basis of cotton fiber quality. They will also benefit cotton molecular breeding to improve STR and MIC with *G. mustelinum* alleles and to ascertain the specific genetic basis of these important traits.

## Materials and methods

### Population development and field evaluation

Three generations of interspecific advanced- backcross populations, namely 21 BC_3_F_2_ families and 12 BC_3_F_2:3_ and BC_3_F_2:4_ families were developed as follows: a *G. hirsutum* acc., PD94042 was crossed with *G. mustelinum* (AD4-8), and then F_1_ plants were independently backcrossed to the *G. hirsutum* parent for three cycles; a total of 21 lineages that produced BC_3_F_1_ plants were self-pollinated and generated 21 BC_3_F_2_ families with size ranging from 127 to 160 plants (totally 3,203 BC_3_F_2_ progenies; Wang et al., 2016a); in addition, 12 BC_3_F_2:3_ and BC_3_F_2:4_ families with size ranging from 130 to 160 lines (totally 1,826 lines) were developed for 12 of the 21 BC_3_F_2_ families with enough seeds (Table S1). The 21 BC_3_F_2_ families were planted in 2006; completely randomized designs were used for the 12 BC_3_F_2:3_ and BC_3_F_2:4_ families with two replicate plots in two years (2008 and 2009) in Tifton, Georgia. All cultural practices were performed as described in Wang et al. (2016a). For the BC_3_F_2_ generation, seed-cotton of all bolls was hand-harvested for each plant; for the BC_3_F_2:3_ and BC_3_F_2:4_ generations, seed-cotton was hand-picked from two random replicate plots as two samples. Seed-cottons were ginned on a saw gin, and then STR and MIC were tested by the Cotton Incorporated Textile Services Laboratory (Cotton Incorporated, Cary, N.C.) by using the Uster High Volume Instrument (HVI; Zellweger-Uster, Knoxville, Tenn.).

### Genotyping and data analysis

The BC_3_F_1_ plants were genotyped with 218 SSR markers selected for even representation of our interspecific *G. mustelinum* by *G. hirsutum* map which comprised 1,055 loci (Wang et al., 2016b), constructed with an F_2_ population of the same two parents. The markers with introgression from *G. mustelinum* in the BC_3_F_1_s were then screened in the corresponding BC_3_F_2_ families for genotyping (Wang et al., 2016a). An average of 58 markers were used to genotype each BC_3_F_2_ family, with marker numbers ranging from 47 to 81, and the size of BC_3_F_2_ families ranged from 127 to 160. This set of genotype data based on each BC_3_F_2_ individual also constitutes the genotype of the corresponding BC_3_F_2:3_ and BC_3_F_2:4_ lines.

For every marker locus that segregated within BC_3_F_2_ families, one-way variance analyses were used to test associations between phenotypes and marker genotypes for statistical significance. The GLM procedure in the SAS ver.8 software package (SAS Institute, 1999) was used to carry out the analyses, and the significance threshold was set at *P* < 0.001 for *F*-test. The gene action modes (additive, a and dominant, d) for individual QTLs were evaluated with their significance levels estimated as described by Paterson et al. (1990). The dominance/additive (d/a) ratio of 3 was used as the threshold to determine whether the QTLs were over- or under-dominant (Chee et al., 2005a).

QTLs were also detected with the software QTLNetwork V2.1 (Yang et al., 2008) for STR and MIC in each of the BC_3_-derived families, so as to map epistatic QTLs and also help confirm the reliability of the QTLs identified by one-way variance analyses. The critical F value calculated based on 1,000 permutation tests was used in the mixed model-based composite interval mapping (MCIM) method, with walk speed and window size set at 1 and 10 cM, respectively. A putative main-effect or epistatic QTL was claimed with the significance threshold set at *P* = 0.001. Considering environmental effects, QTLs were also mapped in joint analysis for the 12 families that were grown in three different environments/generations (BC_3_F_2_, BC_3_F_2:3_, and BC_3_F_2:4_) with the software QTLNetwork V2.1 (Yang et al., 2008). QTLs sharing a common marker between the two methods (QTLNetwork 2.1 and one-way variance analyses) were regarded as the same QTLs. QTL Nomenclature of QTLs was performed as previously described by McCouch et al. (1997); the QTL name began with a “q,” representing a QTL, followed by an abbreviation of the trait name, the chromosome name, and consecutive numbers indicating the QTL number of the same trait on the same chromosome (Wang et al., 2017).

For the loci that segregated in two or more families, two-way mixed model variance analyses were also applied, using the MIXED procedure of the SAS ver. 8 package, including genotype (G) as a fixed factor and family (F) and genotype × family (G × F) interaction as random factors, and the residual maximum likelihood (REML) method was used to estimate model parameters. Marker-trait association (genotype factor) was evaluated with an *F* statistic using a general Satterthwaite approximation for the denominator degrees of freedom (SAS Institute, 1999). A likelihood-ratio (ChiSq, χ^2^) test was carried out for the G × F interaction (Self and Liang, 1987; Chee et al., 2005a). *P* < 0.001 was set as the significance level for both G and G × F effects.

## Results

### Phenotypic distribution and correlations

**Figure 1:**
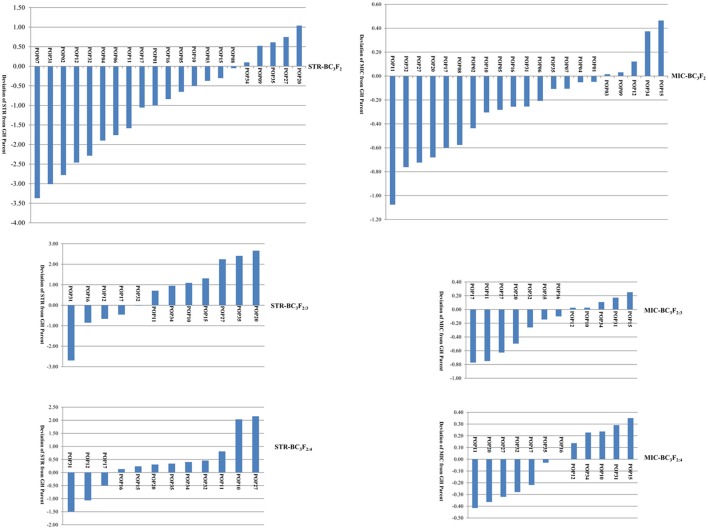
Phenotypic distribution of fiber strength (STR) and fineness (Micronaire, MIC) demonstrated by family mean deviation from recurrent *Gossypium hirsutum* (GH) parent.

The distributions of STR and MIC of the BC_3_ progenies are shown in Figure 1 and Table 1. Both traits expressed significant segregation in the three generations, and their distribution ranges were wider in BC_3_F_2_ than BC_3_F_2:3_ and BC_3_F_2:4_ (Table 1). Although *G. mustelinum*, the wild parent cannot produce spinnable fiber, many BC_3_ progenies show higher STR and lower MIC (usually lower MIC is preferred) than the cultivated parent (*G. hirsutum*, Table 1). For STR, five of 21 BC_3_F_2_, seven of 12 BC_3_F_2:3_, and nine of 12 BC_3_F_2:4_ families showed higher mean values than the *G. hirsutum* parent; four families, namely POP20, POP27, POP34, and POP35 had higher mean values than the *G. hirsutum* parent in all three generations (Figure 1). For MIC, 16 of 21 BC_3_F_2_, seven of 12 BC_3_F_2:3_, and also seven of 12 BC_3_F_2:4_ families showed mean values lower than that of the *G. hirsutum* parent; seven families, namely POP11, POP16, POP17, POP20, POP27, POP32, and POP35 showed lower mean values than the *G. hirsutum* parent in all three generations (Figure 1). Correlations were calculated to evaluate relationships between STR and MIC. The correlation coefficients were −0.005, −0.129, and −0.206 in BC_3_F_2_, BC_3_F_2:3_, and BC_3_F_2:4_ respectively, and the correlations reached significant level of *P* < 0.01 in BC_2_F_2:3_ and BC_3_F_2:4_.

**Table 1:** Summary statistics of fiber strength and fineness measured on the *G. hirsutum* parent and BC_3_ progenies.

**Generation**	**Trait**	**Progeny**			***G.hirsutum***
		**Range**	**Mean ±*SD***	**CV (%)**	**Parent**
BC_3_F_2_	Fiber strength (STR, cN/tex)	17.5–35.3	27.1 ± 2.27	8.4	28.0
	Micronaire (MIC)	2.0–6.6	4.3 ± 0.74	17.2	4.6
BC_3_F_2:3_	Fiber strength (STR, cN/tex)	20.2–41.9	30.5 ± 2.35	7.7	29.9
	Micronaire (MIC)	2.4–5.99	4.5 ± 0.54	12.0	4.7
BC_3_F_2:4_	Fiber strength (STR, cN/tex)	25.4–37.7	31.0 ± 1.63	5.3	30.6
	Micronaire (MIC)	3.3–5.6	4.6 ± 0.38	8.3	4.6

### Main-effect QTLs detected for each trait

**Figure 2:**
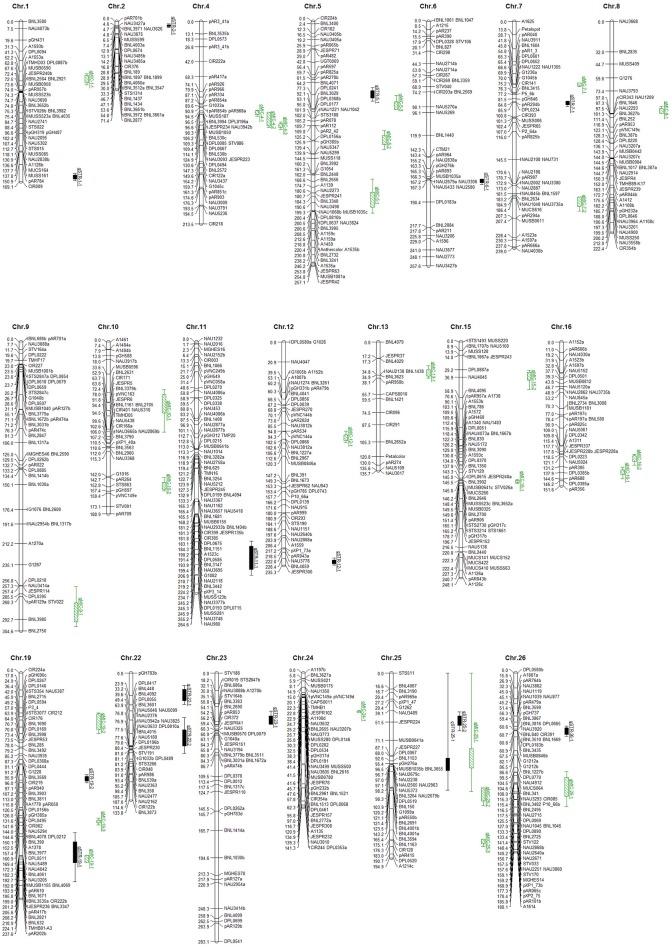
QTLs of fiber strength (STR) and fineness (Micronaire, MIC). The solid black bars indicated QTLs of STR, and the green bars filled with slashes indicated QTLs of MIC.

By assuming that each block of linked markers showing significant marker-trait association (*P* < 0.001) within a family denoted a single QTL, a total of 42 non-overlapping QTLs were identified in the three generations of BC_3_-derived families (Figure 2 and Table 2). These QTLs were mapped to 20 chromosomes, with 22 located on 12 A-subgenome chromosomes and 20 located on eight D-subgenome chromosomes. One or more QTLs for STR and MIC were identified in 13 of the 21 families, with a maximum of five QTLs in each of three families (POP12, POP15, and POP32). Eighteen of the 42 QTLs could be identified at least twice near the same markers in different generations/families or near linked markers in the same family. Twenty-eight of the 42 QTLs were also identified by QTLNetwork. The detailed QTL information is listed in Figure 2 and Table 2.

**Table 2:** Biometrical parameters of QTLs affecting fiber strength and fineness.

**QTL^a^**	**Generation**	**Locus**	**Family**	***R*^2^ (%)^b^**	**A^b^**	**D^b^**	**D/A ratio^b^**	**Mode of action^c^**
qSTR-1-1^*^	BC_3_F_2:3_	CIR009	POP20	12.56	−1.46	−0.80	0.55	A
qSTR-2-1^*^	BC_3_F_2:3_	BNL3971	POP20	12.17	−1.06	−0.08	0.08	A
	BC_3_F_2:3_	DPL0674	POP20	11.14	−1.24	−0.43	0.34	A
qSTR-5-1^*^	BC_3_F_2_	DPL0241	POP04	14.30	−0.13	−1.54	11.51	H
*qSTR-6-1*	BC_3_F_2_	NAU5433	POP32	16.69	1.90	0.71	0.38	A
qSTR-7-1^*^	BC_3_F_2_	DPL0234	POP16	14.04	1.23	0.28	0.23	A
qSTR-11-1^*^	BC_3_F_2_	BNL3442	POP16	18.83	1.40	0.69	0.49	A
	BC_3_F_2:3_	BNL3442	POP16	23.10	1.26	0.01	0.00	A
	BC_3_F_2_	MUSS123b	POP16	11.98	0.98	0.10	0.10	A
	BC_3_F_2:3_	MUSS123b	POP16	29.22	1.45	0.21	0.14	A
	BC_3_F_2:3_	NAU3377b	POP16	18.12	1.66	–	–	–
*qSTR-12-1*	BC_3_F_2_	JESPR300	POP03	16.32	1.47	0.86	0.59	A
qSTR-19-1^*^	BC_3_F_2_	BNL3535a	POP12	12.32	0.80	0.27	0.34	A
	BC_3_F_2:3_	BNL3535a	POP12	11.29	0.73	−0.29	−0.40	A
	BC_3_F_2_	NAU3205	POP12	12.74	0.88	0.42	0.47	A
	BC_3_F_2:3_	NAU3205	POP12	11.70	0.85	−0.09	−0.10	A
	BC_3_F_2:3_	BNL632	POP12	11.88	0.44	−0.82	−1.88	D
	BC_3_F_2:3_	NAU5489	POP12	13.34	0.80	−0.41	−0.52	A
qSTR-19-2^*^	BC_3_F_2_	BNL3811	POP15	8.96	−0.84	0.00	0.00	A
	BC_3_F_2:3_	BNL3811	POP15	11.70	−0.89	−0.41	0.47	A
*qSTR-22-1*	BC_3_F_2_	DPL0417	POP01	18.34	−1.12	−0.13	0.11	A
	BC_3_F_2:3_	DPL0417	POP20	11.61	−1.02	−1.23	1.21	D
qSTR-22-2^*^	BC_3_F_2:3_	CIR048	POP10	10.28	−0.62	−0.08	0.12	A
	BC_3_F_2:3_	DPL0055	POP10	10.80	−0.64	0.06	−0.10	A
	BC_3_F_2:3_	DPL0055	POP32	18.03	−1.51	−0.34	0.22	A
	BC_3_F_2:3_	NAU2376	POP10	12.10	−0.72	−0.21	0.29	A
	BC_3_F_2:3_	NAU2376	POP11	12.03	−0.90	−0.25	0.28	A
qSTR-23-1^*^	BC_3_F_2:3_	BNL3383	POP12	8.74	0.75	0.14	0.18	A
qSTR-25-1^*^	BC_3_F_2_	BNL3264	POP17	14.53	−0.68	0.57	−0.84	A
	BC_3_F_2_	BNL4001b	POP17	14.27	−0.23	1.04	−4.53	H
	BC_3_F_2:4_	BNL4001b	POP12	10.00	−0.27	0.60	−2.22	D
	BC_3_F_2:4_	STS511	POP17	13.22	−0.83	–	–	–
*qSTR-25-2*	BC_3_F_2_	JESPR224	POP02	13.48	−0.21	1.09	−5.24	H
*qSTR-26-1*	BC_3_F_2_	BNL3816	POP16	11.32	1.26	0.46	0.36	A
qMIC-1-1^*^	BC_3_F_2:4_	TMHD03	POP10	16.80	−0.13	0.08	−0.60	A
qMIC-4-1^*^	BC_3_F_2:3_	DPL0196a	POP32	15.58	0.25	−0.37	−1.47	D
	BC_3_F_2:3_	MUSB1050	POP32	21.45	0.41	−0.08	−0.20	A
*qMIC-4-2*	BC_3_F_2:3_	MUSB1050	POP35	10.78	−0.09	−0.27	2.97	D
	BC_3_F_2:3_	DPL0667	POP35	12.26	−0.05	−0.31	5.71	H
qMIC-4-3^*^	BC_3_F_2_	DPL0667	POP17	11.99	0.35	0.14	0.40	A
qMIC-5-1^*^	BC_3_F_2_	BNL2656	POP15	8.92	−0.20	−0.02	0.10	A
	BC_3_F_2:3_	BNL2656	POP15	14.09	−0.21	−0.06	0.30	A
	BC_3_F_2:4_	BNL2656	POP15	7.92	−0.10	−0.02	0.25	A
	BC_3_F_2_	NAU3498	POP15	8.86	−0.21	−0.12	0.56	A
	BC_3_F_2:3_	NAU3498	POP15	20.98	−0.27	−0.14	0.54	A
	BC_3_F_2:4_	NAU3498	POP15	9.70	−0.12	−0.03	0.29	A
	BC_3_F_2:3_	BNL3995	POP15	8.36	−0.16	−0.04	0.29	A
qMIC-5-2^*^	BC_3_F_2_	DPL0156a	POP12	8.35	−0.29	−0.17	0.58	A
qMIC-5-3^*^	BC_3_F_2_	BNL3029	POP34	13.01	−0.45	–	–	–
qMIC-7-1^*^	BC_3_F_2_	NAU1305	POP27	12.31	0.36	0.06	0.17	A
*qMIC-7-2*	BC_3_F_2_	MUCS616	POP32	15.91	0.63	0.26	0.41	A
qMIC-8-1^*^	BC_3_F_2_	CIR343	POP11	16.91	0.59	0.38	0.65	A
qMIC-9-1^*^	BC_3_F_2_	BNL3985	POP11	15.10	0.59	–	–	–
	BC_3_F_2_	DPL0395	POP11	14.54	0.57	–	–	–
qMIC-10-1^*^	BC_3_F_2:3_	BNL2631	POP35	13.00	−0.14	0.18	−1.27	D
	BC_3_F_2:3_	JESPR6	POP35	22.36	−0.15	0.25	−1.67	D
	BC_3_F_2:3_	BNL1161	POP35	16.77	−0.36	–	–	–
	BC_3_F_2:3_	CIR171	POP35	13.25	−0.31	–	–	–
*qMIC-10-2*	BC_3_F_2_	STS863	POP09	9.92	−0.44	–	–	–
*qMIC-12-1*	BC_3_F_2_	DPL0866	POP11	17.45	0.56	0.43	0.77	A
qMIC-13-1^*^	BC_3_F_2_	BNL1438	POP32	16.55	0.48	−0.17	−0.36	A
qMIC-15-1^*^	BC_3_F_2:3_	BNL2646	POP15	10.81	0.16	−0.09	−0.56	A
*qMIC-15-2*	BC_3_F_2_	NAU4045	POP27	13.49	−0.50	–	–	–
*qMIC-16-1*	BC_3_F_2_	DPL0385b	POP27	10.17	0.35	0.12	0.35	A
qMIC-16-2^*^	BC_3_F_2_	NAU5024	POP15	9.12	−0.22	−0.04	0.18	A
	BC_3_F_2:3_	NAU5024	POP15	8.20	−0.16	−0.03	0.21	A
qMIC-19-1^*^	BC_3_F_2_	NAU3205	POP09	17.13	0.11	−0.42	−4.04	H
	BC_3_F_2_	BNL3535a	POP09	13.29	0.17	−0.29	−1.70	D
qMIC-19-2^*^	BC_3_F_2:3_	BNL2715	POP16	14.66	−0.23	−0.07	0.30	A
	BC_3_F_2:3_	CIR212	POP16	13.15	−0.18	−0.20	1.13	D
qMIC-19-3^*^	BC_3_F_2_	BNL3977	POP27	18.21	0.19	0.39	2.02	D
	BC_3_F_2:3_	BNL3977	POP27	12.16	0.13	0.17	1.25	D
*qMIC-24-1*	BC_3_F_2_	NAU3605	POP02	11.15	0.40	0.27	0.68	A
qMIC-25-1^*^	BC_3_F_2_	JESPR224	POP01	18.73	0.16	−0.41	−2.64	D
	BC_3_F_2_	BNL4001b	POP01	19.28	0.83	0.46	0.55	A
	BC_3_F_2_	BNL4001b	POP17	22.95	0.13	−0.53	−3.94	H
	BC_3_F_2_	BNL3264	POP17	20.30	0.14	−0.46	−3.24	H
	BC_3_F_2:3_	BNL3264	POP17	19.50	0.07	−0.31	−4.64	H
	BC_3_F_2:3_	STS511	POP17	10.49	0.27	–	–	–
*qMIC-25-2*	BC_3_F_2:3_	BNL4001b	POP17	22.57	−0.01	−0.41	42.30	H
*qMIC-25-3*	BC_3_F_2:3_	BNL1163	POP10	15.08	0.53	0.69	1.30	D
qMIC-26-1^*^	BC_3_F_2:3_	BNL2725	POP15	8.79	−0.16	−0.05	0.33	A
	BC_3_F_2:3_	BNL341	POP12	11.35	−0.19	−0.07	0.34	A
	BC_3_F_2:3_	BNL341	POP15	9.20	−0.15	0.01	−0.05	A

*Each row corresponds to a one-way analysis of variance for the indicated locus*.

a* * indicating that the QTL was also detected by QTLNetwork*.

b *Quantitative parameters: R^2^, percentage of phenotypic variation explained by the marker genotype at the corresponding marker and family; A, additive, a positive number indicates that the alleles from the G. hirsutum parent increase trait values; a negative number indicates that the alleles from the G. mustelinum parent increase trait values. D, dominance. D/A ratio, overdominance effect*.

c *Modes of gene action are indicated by: A, additivity; D, dominance; H, overdominance. Missing values correspond to dominant marker loci*.

### QTLs for STR

A total of 15 non-overlapping QTLs were identified on 12 chromosomes for STR (Figure 2 and Table 2), with seven located in the A-subgenome and eight located in the D-subgenome. Seven QTLs were found at least twice near the same markers in different generations/families or near linked markers in the same family, namely *qSTR-2-1, qSTR-11-1, qSTR-19-1, qSTR-19-2, qSTR-22-1, qSTR-22-2*, and *qSTR-25-1*. The percentage of variance explained (PVE) by individual QTLs ranged from 8.74% (*qSTR-23-1*) to 29.22% (*qSTR-11-1*), with an average of 13.86%. *G. mustelinum* alleles increased STR for eight of the 15 QTLs (Table 2). Ten of the 15 QTLs could also be identified by the MCIM method of QTLNetwork (Table 2).

### QTLs for MIC

A total of 27 non-overlapping QTLs for MIC were identified on 15 chromosomes with 15 located on nine A-subgenome chromosomes and 12 located on six D-subgenome chromosomes (Figure 2 and Table 2). Eleven QTLs were found at least twice near the same markers in different generations/families or near linked markers in the same family. The PVE of each individual locus ranged from 7.92% (*qMIC-5-1*) to 22.95% (*qMIC-25-1*), with an average of 14.06%. For 15 of the 27 QTLs, *G. mustelinum* alleles reduced MIC and therefore contributed to finer fiber (Table 2). Eighteen of the 27 QTLs could also be identified by the MCIM method of QTLNetwork (Table 2).

### Consistency of QTLs across families

A total of 211 of the 218 SSR marker loci were segregating in two or more families; consequently, two-way ANOVA was performed to identify marker-trait associations, so as to evaluate their consistency among different families. Significant (*P* < 0.001) among-family G effects were identified at 13 and 10 loci for STR and MIC respectively (Table S2). For STR, the 13 loci appear to represent eight non-overlapping genomic regions, and QTLs were identified in four regions in within-family analysis (*qSTR-5-1, qSTR-6-1, qSTR-19-2*, and *qSTR-23-1*). For MIC, the 10 loci represent three non-overlapping genomic regions, for which QTLs were detected in three regions in within-family analysis (*qMIC-5-1, qMIC-7-1*, and *qMIC-16-1*; Table 2, Table S2).

For MIC, a total of five loci were significant (*P* < 0.001) for G × F interactions (Table S3). QTLs were detected at all five loci (*qMIC-4-1, qMIC-4-2, qMIC-10-1, qMIC-15-2, qMIC-19-3*, and *qMIC-25-2*; Table 2, Table S3) in different segregating families. No locus was significant for G × F interactions for STR.

### Epistatic QTLs and their interactions with environments

A total of 13 epistatic QTLs were identified with significant additive × additive (AA) effects (*P* < 0.001), most of which (10/13) involved loci that were not linked to any main-effect QTLs (Table 3). For STR, the interaction between a region on Chr5 and another region on Chr19 was identified in both BC_3_F_2:3_ and joint analysis in POP34 simultaneously. *G. mustelinum* alleles increased STR (with negative AA effects) for three epistatic QTLs, and alleles from *G. mustelinum* decreased MIC (with positive AA effects) for five epistatic QTLs (Table 3). The interactions between epistatic QTLs with environment were listed in Table 3.

**Table 3:** Estimated epistasis and epistasis × environment interaction effects of QTLs for fiber strength and fineness.

**Trait**	**Gen./Env.^a^**	**Family**	**QTL_i_^b^**	**Chrosome**	**Interval_i_**	**QTL_i_^b^**	**Chrosome**	**Interval_j_**	**AA^c^**	***P*-Value**	**h^2^(aa) (%)^d^**	**h^2^(aae) (%)^e^**
STR	BC_3_F_2:3_	POP20	*qSTR-1-1*	Chr1	MUSS161-CIR009	*qSTR-2-1*	Chr2	BNL3971-DPL0674	1.52	0.000000	9.79	
	BC_3_F_2:3_	POP34		Chr5	DPL0241-BNL3029	–	Chr19	CIR212-DPL0444	1.59	0.000151	11.68	
	Joint	POP34		Chr5	DPL0241-BNL3029	–	Chr19	CIR212-DPL0444	1.15	0.000000	7.59	0.54
	Joint	POP31	–	Chr3	BNL3441-BNL3267a	–	Chr19	BNL2715-CIR212	−1.24	0.000013	9.99	1.43
	Joint	POP20	–	Chr16	DPL0501-NAU2862	–	Chr18	BNL193-BNL243	−0.98	0.000000	5.50	0.44
	Joint	POP11	–	Chr20	BNL169-STS3242	–	Chr26	NAU3862-NAU1119	0.99	0.000000	10.25	0.13
	Joint	POP17	*qSTR-25-1*	Chr25	BNL3264-BNL4001b		Chr24	NAU3605-DPL0068	−0.93	0.000000	3.14	0.71
MIC	BC_3_F_2_	POP34		Chr15	BNL1350-BNL2646		Chr5	CIR102-DPL0241	0.41	0.000006	3.68	
	BC_3_F_2_	POP10	–	Chr17	BNL1606-NAU3639	–	Chr19	DPL0444-BNL3903	0.63	0.000001	21.05	
	Joint	POP17	–	Chr2	BNL1434-BNL3972	*qMIC-4-3*	Chr4	DPL0085-DPL0667	−0.20	0.000001	6.41	2.71
	Joint	POP16	–	Chr5	BNL3400-CIR102		Chr24	NAU3605-DPL0068	0.20	0.000026	4.13	2.13
	Joint	POP31	–	Chr8	NAU4900-CIR354b	–	Chr23	BNL3511-DPL0378	−0.27	0.000058	3.72	2.18
	Joint	POP10	–	Chr17	NAU3639-NAU5443	–	Chr19	BNL3903-BNL3811	0.27	0.000000	5.85	1.32
	Joint	POP31	–	Chr23	BNL3511-DPL0378	–	Chr21	BNL2589-NAU3074	0.29	0.000350	1.15	6.42

a *Joint: results obtained based on combined data of the BC_3_F_2_, BC_3_F_2:3_, and BC_3_F_2:4_ generations*.

b *QTL with main effect of locus i or j detected in one-way analysis*.

c *Epistatic effects of the additive × additive interaction. A positive number indicates that the G. hirsutum alleles increase trait values; a negative number indicates that the G. mustelinum alleles increase trait values*.

d *Phenotypic variance explained by additive × additive interaction effects*.

e *Phenotypic variance explained by AA by environment effect*.

## Discussion

Cultivated *Gossypium hirsutum* has a narrow gene pool, having experienced genetic bottlenecks during polyploid formation and divergence from its sister polyploid species, as well as during domestication, dispersal by humans, and scientific breeding. The domestication of a comparatively small subgroup of wild species and, in more recent years, over-exploitation of only a few genetic backgrounds in breeding programs of modern Upland cotton by crossing a few closely-related genotypes repeatedly to develop new cultivars has resulted in a genetically depauperate cotton germplasm. Slow genetic progress in improving fiber productivity and quality is indicative that many favorable alleles have reached fixation in the elite gene pool. The deficiency of genetic variation in current germplasm has enhanced the difficulty for breeders to provide low-cost intrinsic genetic solutions to cotton fiber production such as resistance to abiotic and biotic hazards or new needs in fiber quality or agronomic traits.

Because the narrowing of the cotton genetic base, new sources of genetic variation need to be introduced into the cotton gene pool to ensure future success in breeding new cotton cultivars. As a wild tetraploid cotton species diverged far from *G. hirsutum* (Wendel and Cronn, 2003) but sexually compatible with cultivated cotton, *G. mustelinum* may harbor elite alleles beneficial to the improvement of fiber quality traits in Upland cotton (Alves et al., 2013).

In this research, advanced backcross QTL (AB-QTL) analysis was carried out based on SSR markers and phenotypic data collected from three generations of BC_3_-derived families with introgression from *G. mustelinum*. Phenotypic assessment of the advanced backcross populations indicated significant segregation for STR and MIC, which indicated that both negative and positive alleles existed for each trait in both parents (Table 1, Figure 1).

Since the wild parent, *G. mustelinum* cannot produce spinnable fiber, it is not a surprise to find out that for STR, many BC_3_-derived families (16 of 21 in BC_3_F_2_, five of 12 in BC_3_F_2:3_, and three of 12 in BC_3_F_2:4_) showed mean STR values lower than the recurrent *G. hirsutum* parent, PD94042; for MIC, there were five BC_3_-derived families in each generation having higher mean MIC values or coarser fiber than the recurrent parent. This “negative” transgression, yielding a poorer phenotype than that of the recurrent parent, suggests that interspecific hybridization formed many undesirable new gene combinations. Nonetheless, for STR, five of 21 BC_3_F_2_, seven of 12 BC_3_F_2:3_, and nine of 12 BC_3_F_2:4_ families showed mean values higher than that of the *G. hirsutum* parent, and four families (POP20, POP27, POP34, and POP35) had higher mean values than the *G. hirsutum* parent in all three generations; for MIC, most BC_3_-derived families, namely 16 of 21 BC_3_F_2_, seven of 12 BC_3_F_2:3_, and also seven of 12 BC_3_F_2:4_ families outperformed the recurrent parent with lower MIC (finer fiber: Figure 1), and seven families (POP11, POP16, POP17, POP20, POP27, POP32, and POP35) showed mean values lower than the *G. hirsutum* parent in the three generations, showing good stability across environments. Many individual plants/lines in these families have better STR and MIC traits than those of the recurrent parent (Figure 1). This “positive” transgression, yielding a superior phenotype than that of the recurrent parent, suggests that interspecific hybridization formed some desirable new gene combinations, which was also found in previous reports on introgression of *G. barbadense* (Chee et al., 2005a,b; Draye et al., 2005), *G. tomentosum* (Zhang et al., 2011), and *G. darwinii* (Wang et al., 2012). As suggested by previous research (Jiang et al., 2000), gene transfer between gene pools is a significant consequence of interspecific hybridization, which will increase selectable genetic variation and introduce genes for adaptive traits. These results support the hypothesis that fiber quality improvement for Upland cotton may be accomplished by introgressing elite genes from *G. mustelinum* and other tetraploid cotton species.

A total of 15 and 27 non-overlapping QTLs were mapped in one-way analysis of variance with PVE of 13.86 and 14.06% on average for STR and MIC respectively. The effects of many QTLs showed good reproducibility, with 18 of the 42 QTLs detected at least twice near the same markers in different generations/families or near linked markers in the same family. In addition, 28 of the 42 QTLs were also identified by QTLNetwork (Table 2). The detection of QTLs near different markers at corresponding chromosomal locations or across various generations/families with different methods further supports the likelihood that these QTLs are real.

Alleles from the wild *G. mustelinum* increased STR for 53% (eight) of 15 QTLs and decreased MIC (conferred finer fiber) for 56% (15) of 27 QTLs. These QTLs are of great importance to be deeply exploited to transfer elite genes from *G. mustelinum* into Upland cotton. Efforts to improve STR and MIC are now in progress by constructing near-isogenic lines allowing these alleles from *G. mustelinum* to be more easily manageable in cotton breeding programs.

In addition to main-effect QTLs, epistatic QTLs were identified for STR and MIC using data collected over three different generations (Table 3). The results indicated that both epistatic QTLs and main-effect play key genetic roles in STR and MIC (Tables 2, 3). Epistatic QTLs are more complicated to manage compared to utilization of main-effect alleles, naturally at least twice the difficulty will be encountered in testing and introgressing of epistatic QTLs. Furthermore, it is interesting that the majority of epistatic interactions (10/13) were identified between genetic loci that were not linked to any QTL (Table 3), similar to our previous results derived from fiber length, where 14 of 17 epistatic QTLs involved loci not linked to any main-effect QTLs (Wang et al., 2017), suggesting high complexity of fiber quality inheritance. In joint analysis, effects of epistasis × environment were detected for both STR and MIC (Table 3). Although, generally the PVE of epistasis × environment was smaller than that of epistasis, epistasis × environment interactions may add to the difficulty of breeding.

Since more than one family was often segregating for the same chromosomal segment, it was possible to explore genetic background effects on introgressed chromatin. For STR and MIC, significant (*P* < 0.001) among-family G effects were identified at 13 and 10 loci (Table S2), with four and three loci revealing QTLs in within-family analysis for STR (*qSTR-5-1, qSTR-6-1, qSTR-19-2*, and *qSTR-23-1*) and MIC (*qMIC-5-1, qMIC-7-1*, and *qMIC-16-1*). Some among-family G effects demonstrated good reproducibility, with three of 13 for STR, and two of 10 for MIC detected in different generations (Table S2). A total of five loci were significant (*P* < 0.001) for G × F interactions for MIC (Table S3), with QTLs detected in all the five loci (*qMIC-4-1, qMIC-4-2, qMIC-10-1, qMIC-15-2, qMIC-19-3*, and *qMIC-25-2*; Table 2, Table S3). The most extreme case of G × F interaction was detected at the locus MUSB1050 on Chr4 (Table 2, Table S3). In family POP32, alleles from *G. mustelinum* at this locus conferred a decrease of 0.41 for additive effect in MIC that accounted for 21.45% of PVE. Interestingly, this same locus conferred an additive increase of 0.09 for MIC that accounted for 10.78% of PVE in family POP35; this locus also segregated in family POP10, POP11, and POP27 but showed no significant association with MIC.

This study also adds to prior information on the significant influence of the tetraploid D-subgenome on fiber quality traits, although the D-subgenome was derived from a diploid ancestor without the ability to produce spinnable fiber (Jiang et al., 1998; Chee et al., 2005b; Zhang et al., 2008, 2011). In this current study, among the 42 QTLs affecting STR and MIC traits, the D-subgenome (20) had slightly fewer QTLs than the A-subgenome (22). Considering the QTLs for fiber elongation and fiber length detected in our previous reports (Wang et al., 2016a,b, 2017), 73 QTLs were identified in the D-subgenome, more than the A-subgenome (58), which collectively supports the finding that for fiber quality traits, more QTLs occurred on the D-subgenome than the A-subgenome(Jiang et al., 1998; Paterson et al., 2003).

With this fourth report on QTLs for fiber traits from *G. mustelinum*, this series of papers collectively describes six fiber quality traits investigated in 21 BC_3_F_2_, 12 BC_3_F_2:3_, and 12 BC_3_F_2:4_ families, namely fiber elongation (EL), fiber uniformity index (UI), upper-half mean length (UHM), short fiber content (SFC), STR, and MIC. Mean values of some families outperformed the recurrent *G. hirsutum* parent, PD94042, for each trait in each generation; likewise, many individual plants/lines showed superior fiber quality performance than the *G. hirsutum* parent, showing promise that our goal of introgressing alleles from *G. mustelinum* to improve Upland cotton may work.

Two loci (JESPR224 and BNL3264) may be of special interest in this series of reports. Two QTLs were detected near the locus JESPR224 (*qMIC-25-1* in POP01 in BC_3_F_2_, and *qSTR-25-2* in POP02 in BC_3_F_2_), where *G. mustelinum* alleles decreased MIC and increased STR. Four QTLs (*qUHM-25-1, qSTR-25-1, qMIC-25-1*, and *EL25.1*) were detected near the loci BNL3264 on Chr25 in POP17, for which *G. mustelinum* alleles increased EL, UHM, and STR and decreased MIC (Wang et al., 2016a,b, 2017). These QTLs hold promise for improving cotton fiber elongation, length, strength and fineness simultaneously through marker-assisted selection.

More co-locations of QTLs with both desirable and undesirable effects on different traits were observed. For instance, two QTLs (q*ELO-19-1* and *qSTR-19-2*) were detected in POP15 near the locus BNL3811 on Chr19, for which *G. mustelinum* alleles increased STR but decreased EL; three QTLs (*qMIC-12-1, qSFC-12-2*, and *qUI-12-3*) were detected near the locus DPL0866 on Chr12 in POP11, for which *G. mustelinum* alleles decreased MIC but increased SFC and decreased UI; four QTLs (*qUI-10-1, qSFC-10-2, qMIC-10-1*, and *qELO-10-1*) were detected near the locus JESPR6 on Chr10, for which *G. mustelinum* alleles increased EL but they also increased MIC and SFC and decreased UI (Wang et al., 2016a,b, 2017). The co-location of QTLs with opposite effects on different traits indicates the difficulty of improving diverse traits simultaneously in breeding programs, and may also account for the challenges that have been faced with using exotic germplasm in the absence of DNA marker information.

Building on prior work on *G. barbadense, G. tomentosum*, and *G. darwinii*, QTL mapping involving introgression of *G. mustelinum* alleles offers new allelic variation to the Upland cotton gene pool; in addition, the new germplasm created here offers an opportunity for the cotton community to explore *G. mustelinum* alleles in an elite cultivated background, and also provided materials potentially useful in cotton breeding programs. Wild cotton species represent a repository of divergent and in some cases favorable alleles for a variety of traits including fiber quality. The sixth and seventh tetraploid cotton species found and confirmed, *G. ekmanianum* Wittmack (endemic to the Dominican Republic; Krapovickas and Seijo, 2008; Wendel and Grover, 2015) and *Gossypium* sp. nov. (found from two islands, Wake and Peale in the Wake Atoll in the Pacific Ocean; Wendel and Grover, 2015) offer additional scope for exploration of gene introgression.

## Author contributions

AP conceived and designed the experiments; PC and OM oversaw crossing, phenotyping, and genotyping, which were performed by BW, ZhiZ, ZheZ and EL; DJ oversaw fiber analysis; BW, XD, LS, and TS analyzed the data; BW, AP, and PC wrote the paper.

### Conflict of interest statement

The authors declare that the research was conducted in the absence of any commercial or financial relationships that could be construed as a potential conflict of interest.
