# Supplementary material for: Advanced Backcross QTL Analysis of Fiber Strength and Fineness in a Cross between Gossypium hirsutum and G. mustelinum
Source: Front Plant Sci. 2017 Oct 25;8:1848. doi: 10.3389/fpls.2017.01848 (PMC5661169; doi:10.3389/fpls.2017.01848)
Supplement: Supplementary file 1 [file Table1.DOCX]

**Table S1 Population size and number of SSR loci segregating in BC_3_F_2_, BC_3_F_2:3_ & BC_3_F_2:4_ families**

| Family | Generation (s) | Number of Plants/Lines | Number of  Markers |
| --- | --- | --- | --- |
| POP01 | BC_3_F_2_ | 157 | 61 |
| POP02 | BC_3_F_2_ | 160 | 64 |
| POP03 | BC_3_F_2_ | 160 | 57 |
| POP04 | BC_3_F_2_ | 158 | 50 |
| POP05 | BC_3_F_2_ | 160 | 51 |
| POP06 | BC_3_F_2_ | 160 | 47 |
| POP07 | BC_3_F_2_ | 160 | 58 |
| POP08 | BC_3_F_2_ | 127 | 47 |
| POP09 | BC_3_F_2_ | 135 | 48 |
| POP10 | BC_3_F_2_/BC_3_F_2:3_/BC_3_F_2:4_ | 141 | 51 |
| POP11 | BC_3_F_2_/BC_3_F_2:3_/BC_3_F_2:4_ | 152 | 81 |
| POP12 | BC_3_F_2_/BC_3_F_2:3_/BC_3_F_2:4_ | 152 | 53 |
| POP15 | BC_3_F_2_/BC_3_F_2:3_/BC_3_F_2:4_ | 159 | 53 |
| POP16 | BC_3_F_2_/BC_3_F_2:3_/BC_3_F_2:4_ | 130 | 49 |
| POP17 | BC_3_F_2_/BC_3_F_2:3_/BC_3_F_2:4_ | 157 | 66 |
| POP20 | BC_3_F_2_/BC_3_F_2:3_/BC_3_F_2:4_ | 157 | 70 |
| POP27 | BC_3_F_2_/BC_3_F_2:3_/BC_3_F_2:4_ | 152 | 76 |
| POP31 | BC_3_F_2_/BC_3_F_2:3_/BC_3_F_2:4_ | 160 | 72 |
| POP32 | BC_3_F_2_/BC_3_F_2:3_/BC_3_F_2:4_ | 159 | 56 |
| POP34 | BC_3_F_2_/BC_3_F_2:3_/BC_3_F_2:4_ | 147 | 54 |
| POP35 | BC_3_F_2_/BC_3_F_2:3_/BC_3_F_2:4_ | 160 | 50 |
