# Supplementary material for: Advanced Backcross QTL Analysis of Fiber Strength and Fineness in a Cross between Gossypium hirsutum and G. mustelinum
Source: Front Plant Sci. 2017 Oct 25;8:1848. doi: 10.3389/fpls.2017.01848 (PMC5661169; doi:10.3389/fpls.2017.01848)
Supplement: Supplementary file 2 [file Table2.DOCX]

**Table S2 Biometrical parameters of SSR loci showing significant among-family G effects**

| Trait | No. | Generation | Chromosome | Locus | Among-family  G effect (Pr>F) | Family | n^a^ | Within-family  G effect (Pr>F)^b^ |
| --- | --- | --- | --- | --- | --- | --- | --- | --- |
| STR | 1 | BC_3_F_2_ | Chr05 | DPL0241 | 0.0009 | POP02 | 160 | 0.0511 |
|  |  |  |  |  |  | POP04 | 158 | 0.0002* |
|  |  |  |  |  |  | POP09 | 135 | 0.5510 |
|  |  |  |  |  |  | POP32 | 159 | 0.0054 |
|  |  |  |  |  |  | POP34 | 147 | 0.0570 |
| STR | 2 | BC_3_F_2_ | Chr05 | BNL3029 | 4.06E-09 | POP02 | 160 | 0.0034 |
|  |  |  |  |  |  | POP04 | 158 | 0.0061 |
|  |  |  |  |  |  | POP07 | 160 | 0.4977 |
|  |  |  |  |  |  | POP12 | 152 | 0.0305 |
|  |  |  |  |  |  | POP27 | 152 | 0.1161 |
|  |  |  |  |  |  | POP31 | 160 | 0.0892 |
|  |  |  |  |  |  | POP32 | 159 | 0.0137 |
|  |  |  |  |  |  | POP34 | 147 | 0.1689 |
|  |  |  |  |  |  | POP35 | 160 | 0.1017 |
|  |  | BC_3_F_2:3_ | Chr05 | BNL3029 | 3.90E-04 | POP12 | 152 | 0.0023 |
|  |  |  |  |  |  | POP27 | 152 | 0.6948 |
|  |  |  |  |  |  | POP31 | 160 | 0.5416 |
|  |  |  |  |  |  | POP32 | 159 | 0.4262 |
|  |  |  |  |  |  | POP34 | 147 | 0.0973 |
|  |  |  |  |  |  | POP35 | 160 | 0.0967 |
| STR | 3 | BC_3_F_2_ | Chr05 | DPL0156a | 1.00E-06 | POP02 | 160 | 0.0149 |
|  |  |  |  |  |  | POP12 | 152 | 0.0038 |
|  |  |  |  |  |  | POP31 | 160 | 0.0719 |
|  |  |  |  |  |  | POP32 | 159 | 0.0431 |
|  |  |  |  |  |  | POP34 | 147 | 0.2735 |
|  |  |  |  |  |  | POP35 | 160 | 0.5489 |
| STR | 4 | BC_3_F_2_ | Chr05 | CIR102 | 2.90E-04 | POP12 | 152 | 0.0174 |
|  |  |  |  |  |  | POP16 | 130 | 0.1345 |
|  |  |  |  |  |  | POP31 | 160 | 0.0147 |
|  |  |  |  |  |  | POP34 | 147 | 0.1105 |
| STR | 5 | BC_3_F_2_ | Chr06 | NAU5433 | 1.22E-05 | POP02 | 160 | 0.0743 |
|  |  |  |  |  |  | POP05 | 160 | 0.1015 |
|  |  |  |  |  |  | POP07 | 160 | - |
|  |  |  |  |  |  | POP08 | 127 | 0.2028 |
|  |  |  |  |  |  | POP32 | 159 | 0.0003* |
|  |  |  |  |  |  | POP35 | 160 | 0.1238 |
| STR | 6 | BC_3_F_2_ | Chr07 | NAU1305 | 7.17E-04 | POP16 | 130 | 0.1154 |
|  |  |  |  |  |  | POP27 | 152 | 0.0079 |
| STR | 7 | BC_3_F_2:3_ | Chr07 | NAU2002 | 9.06E-04 | POP11 | 152 | 0.1335 |
|  |  |  |  |  |  | POP12 | 152 | 0.0544 |
|  |  |  |  |  |  | POP15 | 159 | 0.0294 |
|  |  |  |  |  |  | POP27 | 152 | 0.9944 |
| STR | 8 | BC_3_F_2_ | Chr10 | BNL2631 | 0.00096 | POP04 | 158 | - |
|  |  |  |  |  |  | POP10 | 141 | - |
|  |  |  |  |  |  | POP31 | 160 | - |
|  |  |  |  |  |  | POP34 | 147 | - |
|  |  |  |  |  |  | POP35 | 160 | 0.1969 |
| STR | 9 | BC_3_F_2_ | Chr16 | DPL0385b | 8.46E-04 | POP16 | 130 | 0.0960 |
|  |  |  |  |  |  | POP27 | 152 | 0.0091 |
| STR | 10 | BC_3_F_2_ | Chr18 | STS1155a | 0.0009 | POP08 | 127 | 0.6679 |
|  |  |  |  |  |  | POP09 | 135 | 0.1353 |
|  |  |  |  |  |  | POP15 | 159 | 0.0464 |
|  |  |  |  |  |  | POP31 | 160 | 0.4157 |
|  |  |  |  |  |  | POP32 | 159 | 0.0461 |
|  |  |  |  |  |  | POP34 | 147 | 0.6187 |
| STR | 11 | BC_3_F_2_ | Chr19 | BNL3811 | 2.83E-05 | POP10 | 141 | 0.3330 |
|  |  |  |  |  |  | POP15 | 159 | 0.0006* |
|  |  |  |  |  |  | POP20 | 157 | 0.3308 |
|  |  |  |  |  |  | POP34 | 160 | 0.0258 |
|  |  | BC_3_F_2:3_ | Chr19 | BNL3811 | 4.48E-06 | POP10 | 141 | 0.5867 |
|  |  |  |  |  |  | POP15 | 159 | 0.0002* |
|  |  |  |  |  |  | POP20 | 157 | 0.0537 |
|  |  |  |  |  |  | POP34 | 160 | 0.1129 |
| STR | 12 | BC_3_F_2_ | Chr23 | BNL3383 | 7.46E-06 | POP08 | 127 | 0.4340 |
|  |  |  |  |  |  | POP10 | 141 | 0.5576 |
|  |  |  |  |  |  | POP12 | 152 | 0.0083 |
|  |  |  |  |  |  | POP17 | 157 | 0.1481 |
|  |  |  |  |  |  | POP20 | 157 | 0.0014 |
|  |  |  |  |  |  | POP34 | 147 | 0.0135 |
|  |  | BC_3_F_2:3_ | Chr23 | BNL3383 | 1.75E-04 | POP10 | 141 | 0.2604 |
|  |  |  |  |  |  | POP12 | 152 | 0.0005* |
|  |  |  |  |  |  | POP17 | 157 | 0.7853 |
|  |  |  |  |  |  | POP20 | 157 | 0.1015 |
|  |  |  |  |  |  | POP34 | 147 | 0.1275 |
| STR | 13 | BC_3_F_2_ | Chr23 | CIR019 | 0.0006 | POP12 | 152 | 0.0068 |
|  |  |  |  |  |  | POP17 | 157 | 0.1621 |
|  |  |  |  |  |  | POP20 | 157 | 0.0539 |
|  |  |  |  |  |  | POP34 | 147 | 0.4168 |
| MIC | 1 | BC_3_F_2:3_ | Chr05 | BNL2656 | 6.67E-04 | POP12 | 152 | 0.0290 |
|  |  |  |  |  |  | POP15 | 159 | 0.00003* |
|  |  |  |  |  |  | POP32 | 159 | 0.7508 |
|  |  |  |  |  |  | POP35 | 160 | 0.3066 |
| MIC | 2 | BC_3_F_2_ | Chr07 | DPL0234 | 0.0001 | POP11 | 152 | 0.0208 |
|  |  |  |  |  |  | POP16 | 130 | 0.0055 |
| MIC | 3 | BC_3_F_2_ | Chr07 | NAU1305 | 1.96E-05 | POP16 | 130 | 0.0240 |
|  |  |  |  |  |  | POP27 | 152 | 0.0005* |
| MIC | 4 | BC_3_F_2_ | Chr12 | CIR293 | 1.90E-05 | POP01 | 157 | 0.8903 |
|  |  |  |  |  |  | POP03 | 160 | 0.1727 |
|  |  |  |  |  |  | POP08 | 127 | - |
|  |  |  |  |  |  | POP15 | 159 | - |
|  |  |  |  |  |  | POP16 | 130 | 0.2450 |
|  |  |  |  |  |  | POP17 | 157 | 0.0665 |
|  |  |  |  |  |  | POP20 | 157 | 0.1395 |
|  |  |  |  |  |  | POP27 | 152 | 0.0897 |
|  |  |  |  |  |  | POP31 | 160 | 0.9246 |
|  |  |  |  |  |  | POP32 | 159 | 0.1534 |
| MIC | 5 | BC_3_F_2_ | Chr15 | MUSS523c | 1.64E-06 | POP02 | 160 | 0.4902 |
|  |  |  |  |  |  | POP03 | 160 | 0.0962 |
|  |  |  |  |  |  | POP06 | 160 | 0.0893 |
|  |  |  |  |  |  | POP08 | 127 | 0.0242 |
|  |  |  |  |  |  | POP12 | 152 | 0.0127 |
|  |  |  |  |  |  | POP15 | 159 | 0.0251 |
|  |  |  |  |  |  | POP27 | 152 | 0.2918 |
|  |  |  |  |  |  | POP34 | 147 | 0.0435 |
|  |  |  |  |  |  | POP35 | 160 | 0.3177 |
|  |  | BC_3_F_2:3_ | Chr15 | MUSS523c | 8.85E-05 | POP12 | 152 | 0.0283 |
|  |  |  |  |  |  | POP15 | 159 | 0.0129 |
|  |  |  |  |  |  | POP27 | 152 | 0.3361 |
|  |  |  |  |  |  | POP34 | 147 | 0.0523 |
|  |  |  |  |  |  | POP35 | 160 | 0.0960 |
| MIC | 6 | BC_3_F_2_ | Chr15 | BNL1350 | 7.31E-08 | POP11 | 152 | 0.0367 |
|  |  |  |  |  |  | POP12 | 152 | 0.0012 |
|  |  |  |  |  |  | POP15 | 159 | 0.0253 |
|  |  |  |  |  |  | POP34 | 147 | 0.0203 |
|  |  | BC_3_F_2:3_ | Chr15 | BNL1350 | 3.32E-05 | POP11 | 152 | 0.7630 |
|  |  |  |  |  |  | POP12 | 152 | 0.0154 |
|  |  |  |  |  |  | POP15 | 159 | 0.0034 |
|  |  |  |  |  |  | POP34 | 147 | 0.0211 |
| MIC | 7 | BC_3_F_2_ | Chr15 | BNL2700 | 1.75E-04 | POP11 | 152 | 0.0983 |
|  |  |  |  |  |  | POP12 | 152 | 0.0056 |
|  |  |  |  |  |  | POP34 | 147 | 0.0909 |
| MIC | 8 | BC_3_F_2_ | Chr16 | DPL0385b | 6.56E-05 | POP16 | 130 | 0.0315 |
|  |  |  |  |  |  | POP27 | 152 | 0.0006* |
| MIC | 9 | BC_3_F_2_ | Chr22 | DPL0055 | 0.0009 | POP10 | 141 | 0.4628 |
|  |  |  |  |  |  | POP11 | 152 | 0.2206 |
|  |  |  |  |  |  | POP32 | 159 | 0.0093 |
| MIC | 10 | BC_3_F_2:3_ | Chr24 | BNL2772a | 4.11E-04 | POP10 | 141 | 0.0230 |
|  |  |  |  |  |  | POP11 | 152 | 0.2137 |
|  |  |  |  |  |  | POP20 | 157 | 0.1198 |
|  |  |  |  |  |  | POP27 | 152 | 0.0310 |

^a^n: number of individual plants/lines in the family

^b^ * significant at the P<0.001 level
