# Supplementary material for: Advanced Backcross QTL Analysis of Fiber Strength and Fineness in a Cross between Gossypium hirsutum and G. mustelinum
Source: Front Plant Sci. 2017 Oct 25;8:1848. doi: 10.3389/fpls.2017.01848 (PMC5661169; doi:10.3389/fpls.2017.01848)
Supplement: Supplementary file 3 [file Table3.DOCX]

**Table S3 Biometrical parameters of SSR loci showing significant GxF interactions**

| Trait | No. | Generation | Chromosome | Locus | G*F Interaction (Pr>ChiSq) | Family | n^a^ | Within-family G effect (Pr>F)^b^ |
| --- | --- | --- | --- | --- | --- | --- | --- | --- |
| MIC | 1 | BC_3_F_2:3_ | Chr04 | MUSB1050 | 1.18E-04 | POP10 | 141 | 0.4624 |
|  |  |  |  |  |  | POP11 | 152 | 0.0129 |
|  |  |  |  |  |  | POP27 | 152 | 0.6393 |
|  |  |  |  |  |  | POP32 | 159 | 7.22E-05* |
|  |  |  |  |  |  | POP35 | 160 | 0.0009* |
|  | 2 | BC_3_F_2:3_ | Chr10 | JESPR6 | 2.97E-04 | POP11 | 152 | 0.2836 |
|  |  |  |  |  |  | POP15 | 159 | 0.1442 |
|  |  |  |  |  |  | POP17 | 157 | 0.3113 |
|  |  |  |  |  |  | POP31 | 160 | 0.3015 |
|  |  |  |  |  |  | POP34 | 147 | 0.0332 |
|  |  |  |  |  |  | POP35 | 160 | 3.20E-06* |
|  | 3 | BC_3_F_2_ | Chr15 | NAU4045 | 1.98E-04 | POP10 | 141 | 0.8206 |
|  |  |  |  |  |  | POP12 | 152 | 0.1575 |
|  |  |  |  |  |  | POP15 | 159 | 0.0783 |
|  |  |  |  |  |  | POP17 | 157 | 0.2524 |
|  |  |  |  |  |  | POP27 | 152 | 3.97E-05* |
|  |  |  |  |  |  | POP34 | 147 | 0.2834 |
|  |  |  |  |  |  | POP35 | 160 | 0.7044 |
|  |  | BC_3_F_2:3_ | Chr15 | NAU4045 | 4.88E-04 | POP10 | 141 | 0.3753 |
|  |  |  |  |  |  | POP12 | 152 | 0.0513 |
|  |  |  |  |  |  | POP15 | 159 | 0.0929 |
|  |  |  |  |  |  | POP17 | 157 | 0.0095 |
|  |  |  |  |  |  | POP27 | 152 | 0.0045 |
|  |  |  |  |  |  | POP34 | 147 | 0.3655 |
|  |  |  |  |  |  | POP35 | 160 | 0.9858 |
|  | 4 | BC_3_F_2_ | Chr19 | BNL3977 | 3.57E-04 | POP10 | 141 | 0.4770 |
|  |  |  |  |  |  | POP15 | 159 | 0.1867 |
|  |  |  |  |  |  | POP17 | 157 | 0.5594 |
|  |  |  |  |  |  | POP20 | 157 | 0.1329 |
|  |  |  |  |  |  | POP27 | 152 | 7.09E-06* |
|  |  |  |  |  |  | POP35 | 160 | 0.1695 |
|  | 5 | BC_3_F_2:3_ | Chr25 | BNL4001b | 8.74E-04 | POP12 | 152 | 0.0558 |
|  |  |  |  |  |  | POP17 | 157 | 1.29E-06* |

^a^n: number of individual plants/lines in the family

^b^ * significant at the P<0.001 level
